# A phase I trial of the mTOR inhibitor temsirolimus in combination with capecitabine in patients with advanced malignancies

**DOI:** 10.1002/cam4.3672

**Published:** 2021-02-27

**Authors:** Neel D. Trivedi, Samantha Armstrong, Hongkun Wang, Marion Hartley, John Deeken, A. Ruth He, Deepa Subramaniam, Heather Melville, Chris Albanese, John L. Marshall, Jimmy Hwang, Michael J. Pishvaian

**Affiliations:** ^1^ Lombardi Comprehensive Cancer Center Georgetown University Medical Center Washington DC USA; ^2^ The Ruesch Center for the Cure of Gastrointestinal Cancers Lombardi Comprehensive Cancer Center Georgetown University Medical Center Washington DC USA; ^3^ Inova Schar Cancer Institute Inova Health System Falls Church VA USA; ^4^ Levine Cancer Institute Carolinas HealthCare System Charlotte NC USA; ^5^ Department of Oncology Johns Hopkins University School of Medicine SKCC Washington DC USA

**Keywords:** 5‐fluorouracil, capecitabine, colorectal cancer, mTOR, temsirolimus

## Abstract

**Background:**

Temsirolimus is an mTOR antagonist with proven anticancer efficacy. Preclinical data suggest greater anticancer effect when mTOR inhibitors are combined with cytotoxic chemotherapy. We performed a Phase I assessment of the combination of temsirolimus and capecitabine in patients with advanced solid tumors.

**Methods:**

Patients were enrolled in an alternating dose escalation of temsirolimus (at 15 or 25 mg IV weekly) and capecitabine (at 750, 1000, and 1250 mg/m^2^ twice daily) in separate Q2‐week and Q3‐week cohorts. At the recommended Phase II doses (RP2Ds) of temsirolimus and capecitabine (Q2), seven patients were also treated with oxaliplatin (85 mg/m^2^, day 1) to determine triplet combination safety and efficacy.

**Results:**

Forty‐five patients were enrolled and 41 were evaluable for dose‐limiting toxicities (DLTs). The most common adverse events (AEs) were mucositis, fatigue, and thrombocytopenia. The most common grade 3/4 AEs were hypophosphatemia and anemia. Five patients had DLTs, including hypophosphatemia, mucositis, and thrombocytopenia. The RP2Ds were temsirolimus 25 mg +capecitabine 1000 mg/m^2^ (Q2); and temsirolimus 25 mg +capecitabine 750 mg/m^2^ (Q3). Of the 38 patients evaluable for response, one had a partial response (PR) and 19 had stable disease (SD). The overall disease control rate was 52%. Five of the 20 patients with SD/PR maintained disease control for >6 months.

**Conclusions:**

The combination of temsirolimus and capecitabine is safe on both a Q2‐week and a Q3‐week schedule. The combination demonstrated promising evidence of disease control in this highly refractory population and could be considered for testing in disease‐specific phase II trials.

## Graphical abstract

This manuscript describes a Phase I clinical trial of the combination of temsirolimus and capecitabine in the treatment of patients with advanced solid tumors. We determined that this combination is safe on both a Q2‐week and a Q3‐week schedule. Together, temsirolimus and capecitabine also demonstrated promising evidence of disease control in this highly refractory population, and we believe the combination should be considered for testing in disease‐specific phase II trials.

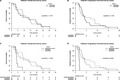

## 1 INTRODUCTION

The mammalian target of rapamycin (mTOR) is a multifunctional serine‐threonine kinase member of the phosphatidylinositol 3′ kinase (PI3K) family.^1^, ^2^ Preclinical work has established that the mTOR/AKT/PI3K signaling pathway is dysregulated in some cancers, leading to uncontrolled cell growth.^3^, ^4^ Rapamycin (also known as sirolimus) is a natural macrolide antibiotic that was discovered ~50 years ago. This agent binds with high affinity to the binding protein FKBP12 to regulate mTOR signaling, thus, inhibiting mRNA synthesis and translation, and resulting in a significant decrease in protein synthesis and cell growth.^5^, ^6^, ^7^

Due to mTOR's action on tumor cell growth, vascular smooth muscle cells, and hypoxia‐inducible factor synthesis, the desired effect of mTOR inhibition is the suppression of tumor cell proliferation and inhibition of angiogenesis, even under hypoxic conditions. Thus, mTOR inhibition has the potential to negatively affect many solid tumors, and because dysregulated mTOR signaling has been seen to occur despite exposure to cytotoxic agents,^8^, ^9^ hence, allowing cell growth in the face of cytotoxicity and under hypoxic conditions, the administration of an mTOR inhibitor together with cytotoxic chemotherapy is an even more attractive therapeutic strategy.^10^, ^11^ Indeed, preclinical studies have shown that mTOR inhibition overcomes platinum resistance in lung and brain cancer cell lines and xenograft models,^12^, ^13^ gemcitabine resistance in pancreatic cancer xenograft models,^14^ and doxorubicin resistance in PTEN‐mutated prostate cancer cell‐lines.^15^

Temsirolimus (Torisel™) is an ester analog of rapamycin and is a targeted anticancer agent that specifically inhibits mTOR.^2^ Temsirolimus is FDA approved for the treatment of renal cell carcinoma,^16^ although it has promising activity in other cancers, including lymphomas^17^, ^18^; breast cancers^19^; endometrial cancers^20^; and neuroendocrine cancers.^21^ In Phase I studies, the combination of an mTOR antagonist plus chemotherapy (as well as targeted therapy) was shown to be safe and suggestive of increased, additive efficacy, compared to chemotherapy alone. Kollmannsberger et al. safely combined temsirolimus, carboplatin, and paclitaxel in patients with a range of solid tumors and demonstrated stable disease (SD) in 46%, and partial response (PR) in 38% of 26 evaluable patients.^22^ In a Phase I study of temsirolimus and 5‐Fluorouracil (5‐FU), Punt et al.^23^ administered temsirolimus in escalating doses (15, 25, 45, and 75 mg) along with a standard regimen of leucovorin (200 mg/m^2^) and 5‐FU IV‐infusion (2600 mg/m^2^) over 24 h.^23^ Treatment was administered once weekly for 6 weeks, followed by a 1‐week rest. The maximally tolerated dose of temsirolimus was defined as 75 mg/m^2^, although fatal mucositis still occurred at doses >25 mg/m^2^.

Capecitabine is a 5‐FU prodrug that has equivalent efficacy to IV 5‐FU (bolus and infusional) and is significantly less toxic than bolus 5‐FU [42]. Being an oral chemotherapeutic agent, capecitabine is more convenient to patients than a lengthy 5‐FU infusion. The FDA approved regimen of 1250 mg/m^2^ capecitabine twice a day (BID) on Days 1–14 of every 3 weeks repeatedly resulted in a stomatitis rate of approximately 20% (all grades) and 2–3% for grades 3 or 4. However, an alternative regimen of 1750 mg/m^2^ BID for 7 days followed by 7 days off, on a 2‐week cycle, resulted in a smaller incidence of stomatitis (9%, all grades; 2%, grade 3).^24^, ^25^ In the only known published comparison of these two schedules, the 7 days on/7 days off regimen was associated with increased time to disease progression.^25^

In this context, and following the lead of Punt et al.,^23^ we initiated a Phase I study of temsirolimus in combination with capecitabine for the treatment of patients with advanced solid malignancies to determine the safest doses of temsirolimus and capecitabine to be used in combination on both a Q2‐week and a Q3‐week schedule. Because mTOR inhibition has been seen to overcome platinum resistance in select solid tumor lines in vitro,^12^, ^13^ and oxaliplatin is often combined with fluoropyrimidine treatment of patients, we included an expansion cohort of added oxaliplatin to investigate the effects of temsirolimus combined, essentially, with XELOX. Survival data for all regimens and schedules were also collected to provide preliminary data to inform future trials.

## 2 METHODS

### 2.1 Patients

Patients had to be ≥18 years of age; have an Eastern Cooperative Oncology Group (ECOG) performance status ≤2; and have acceptable hepatic function (AST and/or ALT ≤2.5× the upper normal limit of institution's normal range [ULN; no liver metastases] or AST and/or ALT <5× ULN [liver metastases], non‐fasting bilirubin ≤1.5× ULN), renal function (serum creatinine ≤1.5× ULN OR creatinine clearance ≥50 mL/min/1.73 m^2^), and bone marrow function (absolute neutrophil count [ANC] ≥1500/mm^3^ [1.5 × 109/L], platelet count ≥100,000/mm^3^ [100 × 109/L], and hemoglobin ≥10.0 g/dL [1.4 mmol/L]). Patients were required to have histologically proven cancer and pathologically confirmed metastases for which there were no known curative therapies, and for which capecitabine (and oxaliplatin, in the oxaliplatin expansion cohort) is approved or compendia listed. Any number of prior lines of therapy were allowed. Patients were enrolled from the Lombardi Comprehensive Cancer Center at Georgetown University, and the study protocol, amendments, and the informed consent forms were approved by the Institutional Review Board at Georgetown University. Investigators obtained informed consent from each participant or participant's guardian prior to screening. The research was conducted in accordance with recognized ethical guidelines, including the Declaration of Helsinki, CIOMS, Belmont Report, and U.S. Common Rule, as described during training in Good Clinical Practice guidelines (CITI Training).

### 2.2 Trial design

**Figure 1:**
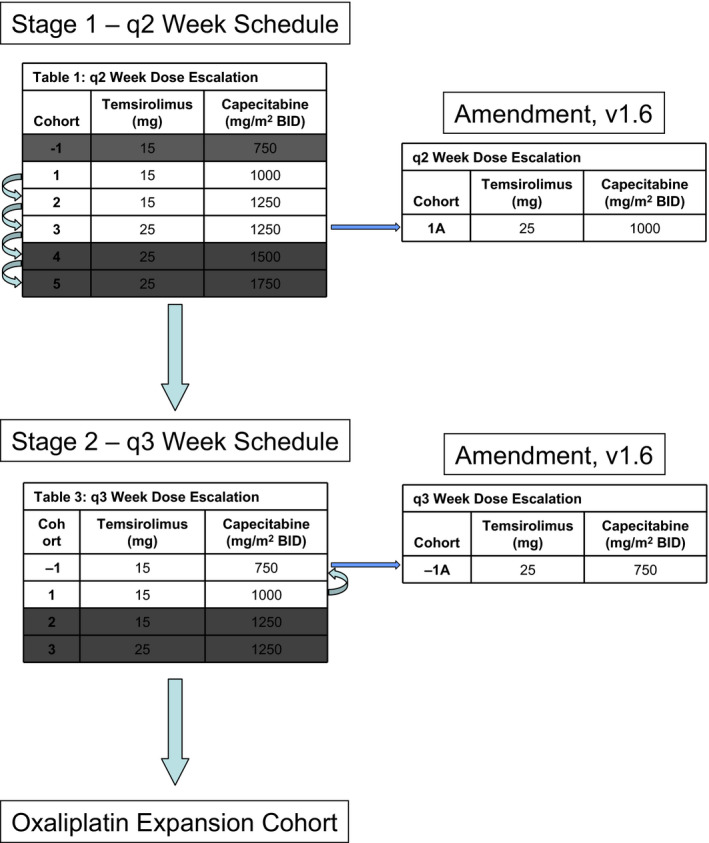
Dose escalation plan for patients treated on study with temsirolimus and capecitabine on a 2‐week and 3‐week schedule. Once discovered, the recommended Phase II dose (RP2D) of temsirolimus plus capecitabine (2‐week schedule) was used in an expansion cohort combined with oxaliplatin (85 mg/m^2^ on day 1 every 2 weeks)

This was an open‐label Phase I study, employing an alternating 3 + 3 dose‐escalation plan (Figure 1). The primary endpoints of the study were to identify the maximally tolerated doses (MTD) and to determine the RP2D of temsirolimus and capecitabine when given as combination therapy. Once the recommended Phase II dose and schedule of temsirolimus plus capecitabine were established, the safety and tolerability of adding oxaliplatin were assessed in an expansion cohort.

During dose escalation, the MTD was defined as the dose level one level below the dose level at which two or more patients out of six experiences a dose‐limiting toxicity (DLT) of one or both agents. A DLT was defined as any of the following events that were possibly or probably related to one or more agents: grade 4 neutropenia lasting greater than 5 days or complicated by fever or infection; grade 4 anemia or thrombocytopenia; grade 3 or 4 non‐hematologic toxicity; and any toxicity, regardless of grade, which resulted in withholding of therapy for >3 weeks. For patients with baseline elevated liver enzymes (AST, ALT, and alkaline phosphatase) due to known intrahepatic metastases, the DLT was defined only as a grade 4 elevation of AST, ALT, or alkaline phosphatase.

#### 2.2.1 Q2‐week schedule

For the 14‐day cycle, the starting dose of temsirolimus was 15 mg IV on Day 1 and 8, and that of capecitabine was 1000 mg/m^2^ PO BID on days 1–7 (Patient Cohort 1; Figure 1). Alternating dose escalation in cohorts of 3 + 3 patients occurred up to a maximal dose of temsirolimus of 25 mg, and a maximal dose of capecitabine of 1250 mg/m^2^. For patients at the 1250 mg/m^2^ dose level of capecitabine who tolerated 15 mg, but not 25 mg of temsirolimus, cohort 1A was established, which enabled dosing with temsirolimus at a dose of 25 mg combined with 1000 mg/m^2^ capecitabine (Figure 1).

#### 2.2.2 Q3‐week schedule

Once the MTD/RP2D was determined for the Q2‐week schedule, a similar dose‐escalation plan was employed for a Q3‐week regimen (Figure 1). The starting doses for the Q3‐week regimen were temsirolimus at 15 mg on days 1, 8, and 15 and capecitabine at 1000 mg/m^2^ BID on days 1–14 of a 21‐day cycle (Patient Cohort 1). However, if intolerable toxicities were observed, temsirolimus was held at 15 mg while the capecitabine dose was reduced to 750 mg/m^2^ (Patient Cohort −1). A cohort was also added of temsirolimus 25 mg and capecitabine at 750 mg/m^2^ BID (Patient Cohort 1A).

#### 2.2.3 Oxaliplatin expansion cohort

Once the Q2‐week MTD/RP2D of temsirolimus plus capecitabine was established, the safety and tolerability of adding oxaliplatin Day 1 of every 2‐week cycle at a dose of 85 mg/m^2^ were also assessed in an expansion cohort.

### 2.3 Patient evaluation

Patients were evaluated weekly until the first response assessment, and then, on day 1 of each treatment cycle thereafter. Radiologic response assessment occurred every 8 weeks for the Q2‐week schedule and every 9 weeks for the Q3‐week schedule. Patients continued to remain on study as long as there was no evidence of progression of disease, and therapy was being adequately tolerated. Information regarding survival and posttreatment therapy was collected every 12 weeks after the final study visit for a period of up to 24 months.

### 2.4 Statistical analysis

Descriptive statistics (*N*, median, and percentage) were used to summarize patient demographics. Adverse events were presented using percentage by cohort and grade according to CTCAE version 3.0. The frequency of adverse events between different cohorts was compared using Fisher's exact test. Kaplan–Meier methodology was used to analyze the time‐to‐event endpoints OS and PFS. Objective response classification followed RECIST criteria and was defined as partial response (PR) or complete response (CR). Clinical benefit was defined as CR + PR + stable disease (SD). The median OS and PFS were estimated with their 95% confidence intervals. Log‐rank test was used to compare the survival experience between different cohorts. SAS software (Version 9.4, SAS Inc.) was used for all the statistical analyses. The primary endpoints of the study were the MTD and RP2D for the temsirolimus/capecitabine combination, determined as described in the *Trial Design* section above. Secondary endpoints included the assessment of objective response rate, median progression‐free survival (PFS), and median overall survival (OS).

## 3 RESULTS

### 3.1 Patients

**Figure 2:**
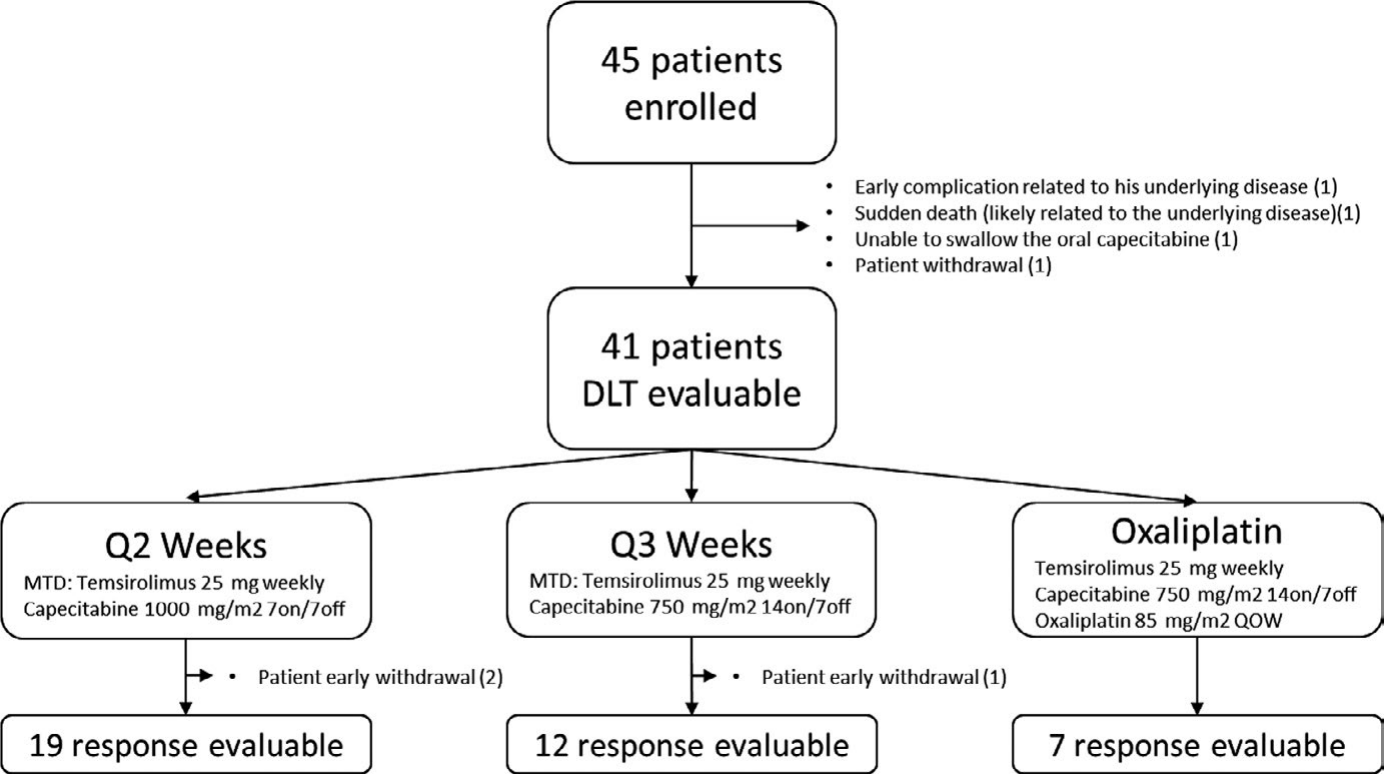
Patient cohort details. In total, 45 patients were enrolled, of which 41 were evaluable for DLT. Of these 41 patients, 21 were treated with temsirolimus and capecitabine on a 2‐week schedule (2 were withdrawn early); 13 on a 3‐week schedule (1 withdrawn); and 7 in the expansion cohort (0 withdrawn). There were 38 response‐evaluable patients on study across the three different cohorts. Survival data were available for all 45 patients

Patient demographics are detailed in Table 1. Forty‐five patients were enrolled into the study between July 2010 and September 2012; 38 into the dose‐escalation portion (23 into the Q2‐week portion and 15 into the Q3‐week portion) and seven into the oxaliplatin expansion cohort (Figure 2, Cohort Details). Twenty‐eight patients were male and 17 were female. The age range was 30–76 years (median, 60 years), and 24 patients had colorectal cancer. Patients received an average of 3.5 prior lines of therapy. Thirty‐six patients had received prior 5‐FU or capecitabine, plus oxaliplatin, while six had not, and three had no specific pretreatment data available. Disease progression was not an eligibility requirement, but most patients had disease progression upon accrual.

**Table 1:** Demographics on all patients enrolled on study (*n* = 45): total and by cohort. Q2 cohort, patients treated with temsirolimus and capecitabine on a 2‐week schedule; Q3 cohort, patients treated with temsirolimus and capecitabine on a 3‐week schedule; expansion cohort, patients treated with temsirolimus and capecitabine plus oxaliplatin on a 2‐week schedule

		All *N* (%)	Q2 Cohort *N* (%)	Q3 Cohort *N* (%)	Expansion Cohort *N* (%)
All patients		45 (100%)	23 (51%)	15 (33%)	7 (16%)
Category	Group				
Age ‐ median		60	67	58	56
Gender	Male	28 (62%)	14 (61%)	8 (53%)	6 (86%)
	Female	17 (38%)	9 (39%)	7 (47%)	1 (14%)
Race	White	33 (73%)	18 (78%)	11 (73%)	4 (57%)
	Black	9 (20%)	3 (13%)	3 (20%)	3 (43%)
	Asian	2 (4%)	2 (9%)		
	Missing	1 (2%)		1 (7 %)	
Primary tumor site	Colorectal	24 (53%)	14 (61%)	7 (47%)	3 (33%)
	Pancreas	9 (20%)	5 (22%)	3 (20%)	1 (14%)
	Lung	3 (7%)	2 (9%)	1 (7%)	
	Appendix	2 (4%)		2 (13%)	
	Bile Duct	1 (2%)			1 (14%)
	Breast	1 (2%)			1 (14%)
	Esophagus	1 (2%)		1 (7%)	
	Jejunum	1 (2%)			1 (14%)
	Kidney	1 (2%)		1 (7%)	
	Other	1 (2%)	1 (4%)		
	Vagina	1 (2%)	1 (4%)		
ECOG	0	15 (33%)	8 (35%)	5 (33%)	2 (29%)
	1	29 (64%)	15 (65%)	9 (60%)	5 (71%)
	2	1 (2%)		1 (7%)	

Four patients were not evaluable for toxicity and efficacy assessment due to early withdrawal from the study. One patient came off study due to a complication related to his underlying disease after just 3 days; one patient died suddenly after just 1 week (related to underlying disease); one patient was unable to swallow the oral capecitabine; and one patient chose to withdraw.

### 3.2 Toxicities and MTD/RP2D

The combination of temsirolimus and capecitabine was adequately tolerated in patients receiving both Q2‐week and Q3‐week regimens. Table 2 details the Adverse Events (AEs) occurring in at least 5% of patients and at least possibly related to therapy. The most common AEs were mucositis/stomatitis, fatigue, and thrombocytopenia. The most common grade 3/4 AEs were hypophosphatemia (*n* = 7) and anemia (*n* = 3). In 28/45 patients, the dose of capecitabine was reduced at some point in treatment. There were DLTs in five patients (one each with hypophosphatemia and mucositis in the Q3‐week cohort; one with a treatment delay due to persistent grade 1 thrombocytopenia in the Q2‐week cohort; one each with hypophosphatemia and mucositis in the oxaliplatin expansion cohort). Adverse event frequency by grade observed in patients on Q2 and Q3‐week regimens (as well as in the oxaliplatin expansion cohort) are shown in Table 3. There was no statistically significant difference in the frequency of adverse events between Q2‐ and Q3‐week dosing of temsirolimus and capecitabine (Fisher test *p* = 0.44), or between Q2‐week, Q3‐week dosing, and the oxaliplatin, temsirolimus, and capecitabine expansion cohort (Fisher test *p *= 0.74).

**Table 2:** Adverse occurring in at least 5% of patients, and at least possibly related to therapy. All 45 patients initially enrolled were evaluable for adverse events. AE, adverse event; CTCAE, Common Terminology Criteria for Adverse Events; Q2 cohort, patients treated with temsirolimus and capecitabine on a 2‐week schedule; Q3 cohort, patients treated with temsirolimus and capecitabine on a 3‐week schedule; expansion cohort, patients treated with temsirolimus and capecitabine plus oxaliplatin on a 2‐week schedule

AE category	CTCAE term	All Patients =41 Number of events (%)			Q2 Cohort *N* = 22 Number of events (%)			Q3 Cohort *N* = 13 Number of events (%)			Expansion Cohort *N* = 6 Number of events (%)		
		All	3,4	1,2	All	3,4	1,2	All	3,4	1,2	All	3,4	1,2
Constitutional	Fatigue	24 (59)	2 (5)	24 (59)	17 (77)	2 (9)	17 (77)	4 (31)		4 (31)	3 (50)		3 (50)
	Anorexia	12 (29)	1 (1)	11 (11)	6 (27)		6 (27)	4 (31)	1 (8)	3 (23)	2 (33)		2 (33)
	Weight Loss	3 (7)		3 (3)	3 (14)		3 (14)						
	Fever In The Absence of Neutropenia	3 (7)		3 (3)	2 (9)		2 (9)	1 (8)		1 (8)			
	Other Pain	3 (7)		3 (3)	2 (9)		2 (9)	1 (8)		1 (8)			
Gastrointestinal	Oral Mucositis/Stomatitis on Clinical Exam	21 (51)	1 (2)	21 (51)	8 (36)		8 (36)	8 (62)		8 (62)	5 (83)	1 (2)	5 (83)
	Symptomatic Oral Mucositis/Stomatitis	15 (37)	2 (5)	15 (37)	7 (32)	1 (5)	7 (32)	7 (54)	1 (8)	7 (54)	1 (17)		1 (17)
	Diarrhea	19 (46)	2 (5)	19 (46)	12 (55)	2 (9)	12 (55)	5 (38)		5 (38)	2 (33)		2 (33)
	Nausea	17 (41)		17 (41)	8 (36)		8 (36)	5 (38)		5 (38)	4 (67)		4 (67)
	Vomiting	5 (12)		5 (12)	2 (9)		2 (9)	1 (8)		1 (8)	2 (33)		2 (33)
	Xerostomia	5 (12)		5 (12)	3 (14)		3 (14)	1 (8)		1 (8)	1 (17)		1 (17)
	Constipation	5 (12)	1 (2)	4 (10)	2 (9)	1 (5)	1 (5)	1 (8)		1 (8)	2 (33)		2 (33)
	Flatulence	4 10)		4 (10)	2 (9)		2 (9	2 (15)		2 (15)			
	Abdominal Pain	4 10)	1 (2)	4 (10)	2 (9)	1 (5)	2 (9)				2 (33)		2 (33)
	Heartburn/Dyspepsia	3 (7)		3 (7)				2 (15)		2 (15)	1 (17)		1 (17)
	Elevated AST	7 (17)	1 (2)	6 (15)	3 (14)	(0)	3 (14)	1 (8)		1 (8)	3 (50)	1 (2)	2 (33)
	Elevated Alkaline Phosphatase	7 (17)		7 (17)	2 (9)		2 (9)	4 (31)		4 (31)	1 (17)		1 (17)
	Elevated ALT	5 (12		5 (12)	3 (14)		3 (14)	1 (8)		1 (8)	1 (17)		1 (17)
	Hyperbilirubinemia	5 (12)		5 (12)	5 (23)		5 (23)						
Hematologic	Thrombocytopenia	21 51)	2 (5)	21 (51)	14 (64)	1 (5)	14 (64)	3 (23)		3 (23)	4 (67)	1 (2)	4 (67)
	Anemia	19 46)	3 (7)	19 (46)	10 (45)	2 (9)	10 (45)	7 (54)	1 (8)	7 (54)	2 (33)		2 (33)
	Leukopenia	17 41)		17 (41)	6 (27)		6 (27)	8 (62)		8 (62)	3 (50)		3 (50)
	Lymphopenia	3 (7)	1 (2)	2 (5)	1 (5)		1 (5)				2 (33)	1 (2)	1 (17)
	Neutropenia	3 (7)		3 (7)	1 (5)		1 (5)				2 (33)		2 (33)
Metabolic	Hypophosphatemia	12 29)	7 (17)	11 (27)	2 (9)	2 (9)	2 (9)	6 (46)	2 (15)	5 (38)	4 (67)	3 (7)	4 (67)
	Hypokalemia	7 17)	1 (2)	7 (17)	4 (18)		4 (18)	1 (8)		1 (8)	2 (33)	1 (2)	2 (33)
	Hypoalbuminemia	6 (15		6 (15)				5 (38)		5 (38)	1 (17)		1 (17)
	Hypocalcemia	5 12)		5 (12)	2 (9)		2 (9)	1 (8)		1 (8)	2 (33)		2 (33)
	Hyperglycemia	3 (7)		3 (7)	2 (9)		2 (9)	1 (8)		1 (8)			
Dermatologic	Rash: Hand‐Foot Skin Reaction	11 27)	1 (2)	11 (27)	5 (23)	1 (5)	5 (23)	4 (31)		4 (31)	2 (33)		2 (33)
	Rash: Acne/Acneiform	10 24)		10 (24)	5 (23)		5 (23)	4 (31)		4 (31)	1 (17)		1 (17)
	Rash: Desquamation	6 (15)		6 (15)	3 (14)		3 (14)	3 (23)		3 (23)			
	Other Rash	4 (10)		4 (10)	3 (14)		3 (14)				1 (17)		1 (17)
	Dry skin	3 (7)		3 (7)	2 (9)		2 (9)	1 (8)		1 (8)			
Pulmonary	Dyspnea	8 (20)	1 (2)	7 (17)	3 (14)	1 (5)	2 (9)	2 (15)		2 (15)	3 (50)		3 (50)
	Cough	5 (12)		5 (12)	1 (5)		1 (5)	3 (23)		3 (23)	1 (17)		1 (17)
Neurologic	Peripheral Neuropathy	7 (17)		7 (17)	1 (5)		1 (5)	3 (23)		3 (23)	3 (50)		3 (50)
	Dizziness	3 (7)		3 (7)	1 (5)		1 (5)				2 (33)		2 (33)
	Headache	3 (7)		3 (7)				2 (15)		2 (15)	1 (17)		1 (17)
Genitourinary	Urinary Frequency/Urgency	3 (7)		3 (7)	1 (5)		1 (5)	1 (8)		1 (8)	1 (17)		1 (17)

**Table 3:** Adverse Events Tables (Q2, Q3, and oxaliplatin expansion cohort). Number of events at each grade and % of the total number of events observed at any grade are displayed for each cohort. These adverse events were possibly, probably, or definitely related to study treatment

Grade	Cohort			Total (*N* = 41)
	Q2 (*N* = 22)	Q3 (*N* = 13)	Expansion (*N* = 6)	
1	215	158	94	467
	70.03%	75.24%	71.21%	
2	62	37	28	127
	20.20%	17.62%	21.21%	
3	24	14	10	48
	7.82%	6.67%	7.58%	
4	3	0	0	3
	0.98%	0%	0%	
5	3	1	0	4
	0.98%	0.48%	0%	
Total	307	210	132	649

Dose escalation in the Q2‐week cohort continued until 2 DLTs were observed at 25 mg of temsirolimus +1250 mg/m^2^ of capecitabine. A protocol amendment allowed for enrollment to 25 mg of temsirolimus (days 1 and 8) +1000 mg/m^2^ of capecitabine (PO BID on days 1–7), which was deemed to be the MTD/RP2D of the Q2‐week cohort. In the first Q3‐week cohort of 15 mg temsirolimus plus 1000 mg/m^2^ capecitabine, there were 2 DLTs. Thus, a protocol amendment allowed for dose escalation of temsirolimus to 25 mg, while capecitabine was administered at a reduced dose of 750 mg/m^2^. This dosing combination was the resulting MTD/RP2D for the Q3‐week cohort: 25 mg temsirolimus (days 1, 8, and 15) plus 750 mg/m^2^ capecitabine (PO BID on days 1–14).

### 3.3 Potential antitumor activity/efficacy

**Figure 3:**
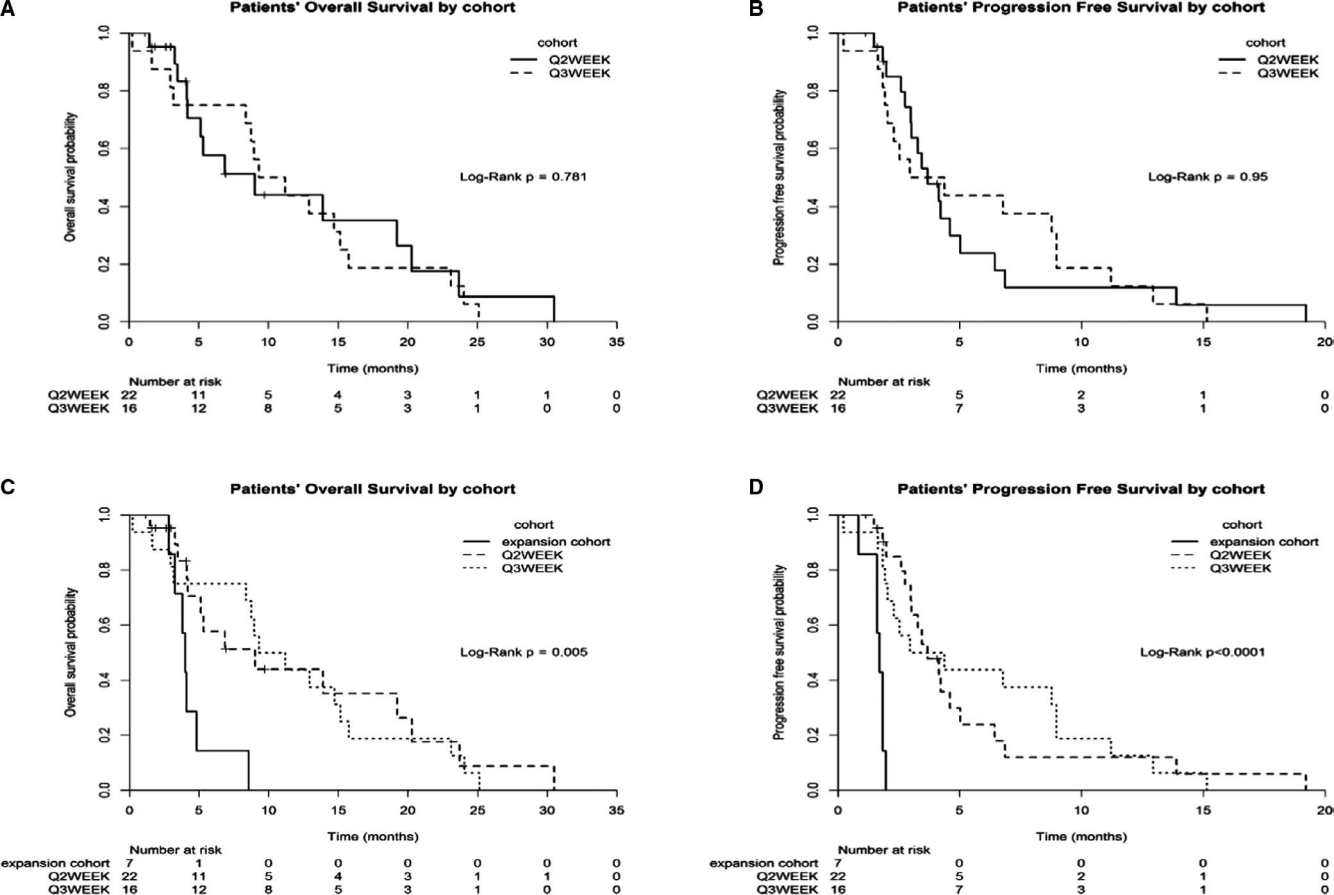
Kaplan‐Meier plots for dose escalation cohorts (Q2‐week and Q3‐week temsirolimus and capecitabine treatment) associated with patient overall survival (A) and progression‐free survival (B), and for both dose escalation (Q2‐week and Q3‐week temsirolimus and capecitabine) and oxaliplatin expansion cohorts associated with patients' overall survival (C) and progression‐free survival (D)

Of the 41 patients evaluable for toxicity, three patients withdrew early due to toxicity. Thus, 38 patients were evaluable for response. There was one confirmed partial response (PR) and 19 patients had stable disease (SD), providing an overall disease control rate of 52%. Five of the 20 patients with SD/PR (25%; 13% of all evaluable patients) maintained disease control for >6 months. There were no differences between the Q2‐week and the Q3‐week administration schedules of temsirolimus and capecitabine with regard to OS (*p* = 0.781) or PFS (*p* = 0.95; Figure 3). However, when oxaliplatin was added to temsirolimus and capecitabine, both the OS and PFS significantly declined (*p* = 0.005 and *p* < 0.0001, respectively; Figure 3). For the oxaliplatin cohort, the PFS was 1.71 months, compared to 3.67 months in the non‐oxaliplatin cohorts (3.68 months for Q2‐week and 3.66 months for Q3‐week); and the OS for the oxaliplatin cohort was 4.01 months compared to 9.61 months in the non‐oxaliplatin cohorts (9.03 months for Q2‐week and 10.25 months for Q3‐week).

As the majority of patients had a diagnosis of colorectal cancer (CRC)—24 of 45 enrolled patients—it seemed appropriate to carry out a separate survival analysis for these patients. Thirteen patients with CRC were on a Q2‐week schedule, eight were on a Q3‐week schedule, and three were part of the oxaliplatin expansion cohort. A comparison of patients indicated that PFS and OS of patients with CRC were similar to those of the general population (*p* > 0.45 for all cohorts).

## 4 DISCUSSION

The combination of temsirolimus and capecitabine was tolerable at our established RP2Ds of temsirolimus 25 mg + capecitabine 1000 mg/m^2^ (on a Q2‐week schedule) and temsirolimus 25 mg + capecitabine 750 mg/m^2^ (Q3), and the toxicities were similar to those reported in the literature following separate temsirolimus or capecitabine.^26^, ^27^, ^28^ Nonetheless, there was clear evidence of enhanced toxicities from the combination because the capecitabine doses had to be reduced for most patients. However, such dose reductions were easily manageable, and patients were able to tolerate the lower doses when reductions were required. The most common grade 3 and 4 toxicities included hypophosphatemia and anemia. Nausea was frequent but remained at grade 1 or 2, and only one patient experienced the high‐grade mucositis observed with higher doses of temsirolimus and 5‐FU.^23^ In the study by Perotti et al,^29^ the rapamycin analog ridaforolimus was administered once a day on days 0, 7, and 14 of a 4‐week cycle in combination with capecitabine administered daily for the first 14 days of a 4‐week cycle. Some of the most common adverse events observed in the Q3‐week cohort of our study were also observed in the Perotti study, although at a slightly higher rate in the Perotti study (mucositis, 69% vs. 54%; anemia, 72% vs. 54%; and leukopenia, 69% vs. 62%). However, thrombocytopenia was not a significant adverse event in our study (23% vs. 62% in the Perotti study), and hypertriglyceridemia was not observed at all (0% vs. 62%). The two studies shared the DLT of mucositis.^29^

With regard to efficacy, in this highly treatment‐refractory patient population, the combination of temsirolimus and capecitabine demonstrated a promising 52% disease control rate (with 13% of patients overall exhibiting disease control for ≥6 months). This efficacy is very similar to the 59% disease control observed by Perotti et al.,^29^ again supporting the concept of further investigation in disease‐specific Phase II trials. There was no apparent difference in efficacy between the Q2‐week and Q3‐week schedules (OS, *p* = 0.78; PFS, *p* = 0.95) for all patients in our study (Figure 3A,B).

Oxaliplatin is often combined with fluoropyrimidine treatment, particularly in GI cancers. In our expansion cohort, we sought to investigate the effects of temsirolimus combined, essentially, with XELOX. When oxaliplatin was added to the temsirolimus/capecitabine regimen, we were surprised to observe that both OS and PFS rates significantly declined (*p* = 0.005 and *p* < 0.0001, respectively; Figure 3C,D) compared with the temsirolimus/capecitabine Q2‐week and Q3‐week regimens, despite no significant worsening of adverse events due to oxaliplatin. Therefore, this triple combination cannot be recommended for further investigation.

The mTOR pathway plays a significant role in the growth of gastrointestinal (GI) cancers, including esophageal, gastric, pancreatic, and colon cancers,^30^, ^31^ and fluoropyrimidines form the backbone of many chemotherapy regimens for patients with GI cancers, making the combination of an mTOR inhibitor with 5‐FU a natural choice for combination therapy trials. Out of the 45 patients recruited into our study, over 50% (24 patients) had CRC. In preclinical studies, mTOR was shown to be an effective target for CRC treatment. Kaneko et al. evaluated the antitumor effect of temsirolimus in CRC cell lines (CaR‐1, HT‐29, and Colon26) in vitro and in vivo in a mouse subcutaneous tumor model.^32^ Temsirolimus inhibited the growth of tumors in all cell lines in all scenarios, and analyses showed that inhibition occurred not only through direct growth inhibition, but also via an antiangiogenic effect.^32^ In 2016, He et al. studied the mechanisms of mTOR inhibitor antitumor activity in CRC cells and xenografts and demonstrated the proapoptotic activity and an essential role of death receptor‐mediated apoptosis on inhibition of 4E‐BP1 phosphorylation.^33^ Finally, Wagner et al. demonstrated that rapamycin combined with 5‐FU or oxaliplatin showed superior tumor suppression compared with rapamycin alone.^34^ Clinically, McRee et al. carried out a Phase I study of everolimus with mFOLFOX6 in the treatment of patients with refractory mCRC.^35^ Everolimus was well‐tolerated, and median OS for all evaluable patients on this mTOR inhibitor plus 5‐FU/LV was a promising 6.9 months. In this context, we performed a separate survival analysis of the 24 CRC patients in our study and compared results with those obtained from patients with all other solid tumors. There were no significant differences in median OS and PFS between patients with CRC and all other patients, regardless of dosing schedule. In the Q2‐week cohort, the median OS rate for patients with CRC was 13.9 months (*n* = 13) versus 6.9 months for non‐CRC patients (*n* = 9) but the confidence intervals in each group were almost identical. Most solid tumors harbored by patients in this study have previously demonstrated response to mTOR inhibition so a difference between subgroups of patients should probably not be expected. Still, a larger study of CRC‐only patients would be warranted.

In conclusion, the combination of temsirolimus and capecitabine is safe on both a Q2‐week and a Q3‐week schedule at the established RP2Ds. The combination demonstrated some promising evidence of disease control in this highly refractory population and should be tested in disease‐specific phase II trials. The addition of oxaliplatin to this combination resulted in worse survival outcomes and is not recommended.

## CONFLICT OF INTEREST

Michael J. Pishvaian: Speaker/Consultant: AstraZeneca/MedImmune, Halozyme, Merck, and Sirtex Medical. Travel, Accommodations, and Expenses Support: AstraZeneca/MedImmune, Merck, and Sirtex Medical. Stock: Perthera. Research Funding to the Institution: Bavarian Nordic, MedImmune; Neel D. Trivedi: No disclosures to report related to this work; Samantha Armstrong: No disclosures to report related to this work; Hongkun Wang: No disclosures to report related to this work; Marion Hartley: No disclosures to report related to this work; John Deeken: No disclosures to report related to this work; A. Ruth He: No disclosures to report related to this work; Deepa Subramaniam: No disclosures to report related to this work; Heather Melville: No disclosures to report related to this work; Chris Albanese: No disclosures to report related to this work; John L Marshall: No disclosures to report related to this work; Jimmy Hwang: No disclosures to report related to this work.

## AUTHOR CONTRIBUTIONS

Michael J. Pishvaian: Planning, conduct, and reporting of the work described in the article; responsible for the overall content as guarantor; Neel D. Trivedi: Conduct and reporting of the work described in the article; Samantha Armstrong: Conduct and reporting of the work described in the article; Hongkun Wang: Conduct and reporting of the work described in the article; Marion Hartley: Conduct and reporting of the work described in the article; John Deeken: Conduct and reporting of the work described in the article; A. Ruth He: Conduct and reporting of the work described in the article; Deepa Subramaniam: Conduct and reporting of the work described in the article; Heather Melville: Conduct and reporting of the work described in the article; Chris Albanese: Conduct and reporting of the work described in the article; John L. Marshall: Conduct and reporting of the work described in the article; Jimmy Hwang: Conduct and reporting of the work described in the article.

## Data Availability

Data are available from the corresponding author on reasonable request.

## References

[1] Abraham RT , Gibbons JJ . The mammalian target of rapamycin signaling pathway: twists and turns in the road to cancer therapy. Clin Cancer Res. 2007;13:3109‐3114. 17545512 10.1158/1078-0432.CCR-06-2798

[2] Rini BI . Temsirolimus, an inhibitor of mammalian target of rapamycin. Clin Cancer Res. 2008;14:1286‐1290. 18316545 10.1158/1078-0432.CCR-07-4719

[3] Zhang Y , Bao C , Mu Q , et al. Reversal of cisplatin resistance by inhibiting PI3K/Akt signal pathway in human lung cancer cells. Neoplasma. 2016;63:362‐370. 26925782 10.4149/304_150806N433

[4] Gong T , Cui L , Wang H , Wang H , Han N . Knockdown of KLF5 suppresses hypoxia‐induced resistance to cisplatin in NSCLC cells by regulating HIF‐1alpha‐dependent glycolysis through inactivation of the PI3K/Akt/mTOR pathway. J Transl Med. 2018;16:164. 29898734 10.1186/s12967-018-1543-2 PMC6000925

[5] Garber K . Rapamycin may prevent post‐transplant lymphoma. J Natl Cancer Inst. 2001;93:1519. 11604471 10.1093/jnci/93.20.1519

[6] Hoeffer CA , Tang W , Wong H , et al. Removal of FKBP12 enhances mTOR‐raptor interactions, LTP, memory, and perseverative/repetitive behavior. Neuron. 2008;60:832‐845. 19081378 10.1016/j.neuron.2008.09.037 PMC2630531

[7] Laplante M , Sabatini DM . mTOR signaling at a glance. J Cell Sci. 2009;122:3589‐3594. 19812304 10.1242/jcs.051011 PMC2758797

[8] Yecies JL , Manning BD . mTOR links oncogenic signaling to tumor cell metabolism. J Mol Med (Berl). 2011;89:221‐228. 21301797 10.1007/s00109-011-0726-6

[9] Mossmann D , Park S , Hall MN . mTOR signalling and cellular metabolism are mutual determinants in cancer. Nat Rev Cancer. 2018;18:744‐757. 30425336 10.1038/s41568-018-0074-8

[10] Atkins MB , Hidalgo M , Stadler WM , et al. Randomized phase II study of multiple dose levels of CCI‐779, a novel mammalian target of rapamycin kinase inhibitor, in patients with advanced refractory renal cell carcinoma. J Clin Oncol. 2004;22:909‐918. 14990647 10.1200/JCO.2004.08.185

[11] Frost P , Moatamed F , Hoang B , et al. In vivo antitumor effects of the mTOR inhibitor CCI‐779 against human multiple myeloma cells in a xenograft model. Blood. 2004;104:4181‐4187. 15304393 10.1182/blood-2004-03-1153

[12] Wu C , Wangpaichitr M , Feun L , et al. Overcoming cisplatin resistance by mTOR inhibitor in lung cancer. Mol Cancer. 2005;4:25. 16033649 10.1186/1476-4598-4-25 PMC1181826

[13] Geoerger B , Kerr K , Tang CB , et al. Antitumor activity of the rapamycin analog CCI‐779 in human primitive neuroectodermal tumor/medulloblastoma models as single agent and in combination chemotherapy. Cancer Res. 2001;61:1527‐1532. 11245461

[14] Ito D , Fujimoto K , Mori T , et al. In vivo antitumor effect of the mTOR inhibitor CCI‐779 and gemcitabine in xenograft models of human pancreatic cancer. Int J Cancer. 2006;118:2337‐2343. 16331623 10.1002/ijc.21532

[15] Grunwald V , DeGraffenried L , Russel D , Friedrichs WE , Ray RB , Hidalgo M . Inhibitors of mTOR reverse doxorubicin resistance conferred by PTEN status in prostate cancer cells. Cancer Res. 2002;62:6141‐6145. 12414639

[16] Hudes G , Carducci M , Tomczak P , et al. Temsirolimus, interferon alfa, or both for advanced renal‐cell carcinoma. N Engl J Med. 2007;356:2271‐2281. 17538086 10.1056/NEJMoa066838

[17] Hess G , Herbrecht R , Romaguera J , et al. Phase III study to evaluate temsirolimus compared with investigator's choice therapy for the treatment of relapsed or refractory mantle cell lymphoma. J Clin Oncol. 2009;27:3822‐3829. 19581539 10.1200/JCO.2008.20.7977

[18] Smith SM , van Besien K , Karrison T , et al. Temsirolimus has activity in non‐mantle cell non‐Hodgkin's lymphoma subtypes: The University of Chicago phase II consortium. J Clin Oncol. 2010;28:4740‐4746. 20837940 10.1200/JCO.2010.29.2813 PMC3020703

[19] Chan S , Scheulen ME , Johnston S , et al. Phase II study of temsirolimus (CCI‐779), a novel inhibitor of mTOR, in heavily pretreated patients with locally advanced or metastatic breast cancer. J Clin Oncol. 2005;23:5314‐5322. 15955899 10.1200/JCO.2005.66.130

[20] Oza AM , Elit L , Tsao M‐S , et al. Phase II study of temsirolimus in women with recurrent or metastatic endometrial cancer: a trial of the NCIC Clinical Trials Group. J Clin Oncol. 2011;29:3278‐3285. 21788564 10.1200/JCO.2010.34.1578 PMC3158598

[21] Duran I , Kortmansky J , Singh D , et al. A phase II clinical and pharmacodynamic study of temsirolimus in advanced neuroendocrine carcinomas. Br J Cancer. 2006;95:1148‐1154. 17031397 10.1038/sj.bjc.6603419 PMC2360568

[22] Kollmannsberger C , Hirte H , Siu LL , et al. Temsirolimus in combination with carboplatin and paclitaxel in patients with advanced solid tumors: a NCIC‐CTG, phase I, open‐label dose‐escalation study (IND 179). Ann Oncol. 2012;23:238‐244. 21447615 10.1093/annonc/mdr063 PMC8890459

[23] Punt CJ , Boni J , Bruntsch U , Peters M , Thielert C . Phase I and pharmacokinetic study of CCI‐779, a novel cytostatic cell‐cycle inhibitor, in combination with 5‐fluorouracil and leucovorin in patients with advanced solid tumors. Ann Oncol. 2003;14:931‐937. 12796032 10.1093/annonc/mdg248

[24] Oh SC , Sur HY , Sung HJ , et al. A phase II study of biweekly dose‐intensified oral capecitabine plus irinotecan (bXELIRI) for patients with advanced or metastatic gastric cancer. Br J Cancer. 2007;96:1514‐1519. 17473829 10.1038/sj.bjc.6603752 PMC2359951

[25] Scheithauer W , Kornek GV , Raderer M , et al. Randomized multicenter phase II trial of two different schedules of capecitabine plus oxaliplatin as first‐line treatment in advanced colorectal cancer. J Clin Oncol. 2003;21:1307‐1312. 12663719 10.1200/JCO.2003.09.016

[26] Scheithauer W , McKendrick J , Begbie S , et al. Oral capecitabine as an alternative to i.v. 5‐fluorouracil‐based adjuvant therapy for colon cancer: safety results of a randomized, phase III trial. Ann Oncol. 2003;14:1735‐1743. 14630678 10.1093/annonc/mdg500

[27] Schmoll H‐J , Cartwright T , Tabernero J , et al. Phase III trial of capecitabine plus oxaliplatin as adjuvant therapy for stage III colon cancer: a planned safety analysis in 1,864 patients. J Clin Oncol. 2007;25:102‐109. 17194911 10.1200/JCO.2006.08.1075

[28] Twelves C , Wong A , Nowacki MP , et al. Capecitabine as adjuvant treatment for stage III colon cancer. N Engl J Med. 2005;352:2696‐2704. 15987918 10.1056/NEJMoa043116

[29] Perotti A , Locatelli A , Sessa C , et al. Phase IB study of the mTOR inhibitor ridaforolimus with capecitabine. J Clin Oncol. 2010;28:4554‐4561. 20855840 10.1200/JCO.2009.27.5867

[30] Buck E , Eyzaguirre A , Haley JD , Gibson NW , Cagnoni P , Iwata KK . Inactivation of Akt by the epidermal growth factor receptor inhibitor erlotinib is mediated by HER‐3 in pancreatic and colorectal tumor cell lines and contributes to erlotinib sensitivity. Mol Cancer Ther. 2006;5:2051‐2059. 16928826 10.1158/1535-7163.MCT-06-0007

[31] Lang SA , Gaumann A , Koehl GE , et al. Mammalian target of rapamycin is activated in human gastric cancer and serves as a target for therapy in an experimental model. Int J Cancer. 2007;120:1803‐1810. 17230506 10.1002/ijc.22442

[32] Kaneko M , Nozawa H , Hiyoshi M , et al. Temsirolimus and chloroquine cooperatively exhibit a potent antitumor effect against colorectal cancer cells. J Cancer Res Clin Oncol. 2014;140:769‐781. 24619662 10.1007/s00432-014-1628-0 PMC11824085

[33] He K , Zheng X , Li M , Zhang L , Yu J . mTOR inhibitors induce apoptosis in colon cancer cells via CHOP‐dependent DR5 induction on 4E‐BP1 dephosphorylation. Oncogene. 2016;35:148‐157. 25867072 10.1038/onc.2015.79 PMC4603992

[34] Wagner M , Roh V , Strehlen M , et al. Effective treatment of advanced colorectal cancer by rapamycin and 5‐FU/oxaliplatin monitored by TIMP‐1. J Gastrointest Surg. 2009;13:1781‐1790. 19565301 10.1007/s11605-009-0948-x

[35] McRee AJ , Davies JM , Sanoff HG , et al. A phase I trial of everolimus in combination with 5‐FU/LV, mFOLFOX6 and mFOLFOX6 plus panitumumab in patients with refractory solid tumors. Cancer Chemother Pharmacol. 2014;74:117‐123. 24819684 10.1007/s00280-014-2474-0 PMC4517671

